# Privacy-by-Design Environments for Large-Scale Health Research and Federated Learning from Data

**DOI:** 10.3390/ijerph191911876

**Published:** 2022-09-20

**Authors:** Peng Zhang, Maged N. Kamel Boulos

**Affiliations:** 1 Data Science Institute & Department of Computer Science, Vanderbilt University, Nashville, TN 37240, USA; 2 Faculdade de Medicina, Universidade de Lisboa, 1649-028 Lisbon, Portugal

**Keywords:** trusted research environments, personal health trains, privacy by design

## Abstract

This article offers a brief overview of ‘privacy-by-design (or data-protection-by-design) research environments’, namely Trusted Research Environments (TREs, most commonly used in the United Kingdom) and Personal Health Trains (PHTs, most commonly used in mainland Europe). These secure environments are designed to enable the safe analysis of multiple, linked (and often big) data sources, including sensitive personal data and data owned by, and distributed across, different institutions. They take data protection and privacy requirements into account from the very start (conception phase, during system design) rather than as an afterthought or ‘patch’ implemented at a later stage on top of an existing environment. TREs and PHTs are becoming increasingly important for conducting large-scale privacy-preserving health research and for enabling federated learning and discoveries from big healthcare datasets. The paper also presents select examples of successful TRE and PHT implementations and of large-scale studies that used them.

## 1. Introduction

This feature review briefly covers some of the most significant, rather than the total, literature, and developments regarding ‘privacy-by-design (or data-protection-by-design) research environments’, namely Trusted Research Environments (TREs, most commonly used in the United Kingdom) and Personal Health Trains (PHTs, most commonly used in mainland Europe). It complements an earlier article by Kamel Boulos et al. published in January 2022 that offered a state-of-the-art summary of a range of privacy-preserving methods and solutions for health research involving disaggregate data about individuals, such as synthetic data generation [1].

### 1.1. Challenges in Health and Healthcare Data Sharing

The rapid development in computing and information technologies has contributed to the exponential growth of data. Much more accurate and timely decision-making is made possible because of this growth. The availability of data has been particularly crucial to the healthcare sector because data understanding and analyses can lead to more accurate diagnoses, effective treatment discoveries, and medical innovations that benefit the entirety of humankind. Healthcare organisations and practices are accumulating innumerable and richly complex patient and care data, which, if joined widely and analysed in-depth, can help generate insights at large-population levels and improve the overall quality of care. However, the very complexity and sensitive private nature of personal medical information have made it difficult for effective data sharing to routinely take place between participants in the sector.

Systems such as Electronic Health Records (EHRs) are widely adopted by hospitals and medical practices to store data about patients, such as vital signs, care notes and summaries, test results, prescriptions, and other communications. These systems are designed to comply with security regulations and data confidentiality agreements, which often results in a number of challenges in relation to healthcare data and their optimal use: (i) data are fragmented as a result of being stored in disparate systems without the ease or the incentive of integration; (ii) medical communication is delayed as medical notes may be missing, inaccessible, or are presented out of context; (iii) access to EHR or other relevant information may be limited or unavailable, creating barriers for diagnosis and necessary medical communication; (iv) data are often non-standardised, or even unstructured, requiring significant effort to sort through and interpret; (v) concerns around data security, privacy and intellectual property place constraints on secondary uses of data, which negatively affects the statistical power of deeper analyses [2,3].

In current systems, data custodians are reluctant to exchange data with other parties quickly or at all due to the severe consequences of breaching existing regulations or privacy agreements [4,5]. As a result, data sharing tends to follow a data release model [6], where many protective procedures are in place to safeguard against insecure, unauthorised and unethical access to a minimised set of data upon request. What makes this model a ‘release’ model is that once shared data leave the original system, they are no longer solely owned by that system. Furthermore, data in their raw form, such as those coming directly from EHRs, might fail to effectively communicate relevant insights in a timely manner, as various sets of essential data might be structured differently, might lack contextual information [2], or might not be directly suitable for sharing [7].

### 1.2. Overcoming Data Sharing Challenges Using TREs and PHTs

To overcome the data-sharing challenges faced by healthcare providers and clinical researchers, the National Health Service in England (NHS) has invested considerably in adopting and developing TREs for the healthcare sector. These TREs are designed as controlled virtual environments for securely storing healthcare data, enabling analysts and researchers to conglomerate and share data without revealing any personally identifiable information. PHTs are a related development pioneered in mainland Europe in which research questions, like a train, ‘travel’ to the data (securely stored in ‘data stations’) rather than data from various sources having to be transported (or externally released) to research questions, ensuring that sensitive data never leave their providing institutions (or ‘stations’) [8].

With access to more comprehensive datasets, in-depth analyses and studies can now be safely performed on large-scale, population-level data involving millions of patient records. The general approach adopted by these environments models a data access system that is fundamentally different from the data release system in that owners of the shared data remain in full control of their data and may grant only approved personnel within the environment access to available, anonymised data [6]. Successful implementation and adoption of TREs and PHTs rely on public trust and consent from patients since uninformed access to data, such as data being exploited by marketers or insurance companies, raises ethical issues and privacy concerns and eventually erodes public trust. In response to these concerns, thought leaders and designers of these environments have proposed tracking and auditing data access activities, in addition to the assurance that disclosive information is properly shielded [6,9].

The rest of this article presents a brief overview of TREs and PHTs in the healthcare sector, covering their privacy-by-design (or data-protection-by-design) principles, main components and benefits, with select examples of successful implementations of these environments and of large-scale studies that used them.

## 2. Key Aspects and Components of TREs

This section details key stakeholders and principles of TREs, their technical components, and how the components interact together to form a data access system with a shared governance that maintains the confidentiality and privacy of patient data.

### 2.1. Key Stakeholders

Formed by healthcare data providers, custodians, and curators, the UK Health Data Research Alliance (UKHDRA) proposed TREs as an approach to provide access to data and data analytics to enhance healthcare research while addressing the needs and concerns of key stakeholders, who must always be involved for the successful adoption of TREs. The identified stakeholders and their roles are as follows [9].

- **Citizens and patients** are not primary users of TREs, but they collectively contribute a wealth of data that help inform and improve public health, in which case they also serve as the beneficiary. When a new system is proposed, the concern of data privacy naturally arises, so advocators of TREs will be expected to earn patients’ trust by being in continual and transparent communication with them.
- **Researchers and analysts** are the intended users of TREs who will request access to data that meet their research requirements. It is also important to satisfy users’ need for analytical tools that can securely support their data processing and data science tasks.
- **Data custodians** are managers, key providers, and curators of patient data who remain in control of their data even when others are granted access to these data. The provision and hosting of data may be outsourced to third-party TRE providers, but only data custodians maintain ownership of their data.
- **Funding agencies** provide financial support to create technical prototyping and development of TREs, with an interest in pushing innovative and effective healthcare research.

### 2.2. Five Safes Framework

UKHDRA adopted a modified version of the ‘Five Safes’ framework created by the United Kingdom Office for National Statistics (ONS) [10] and proposed an optional extension to it to serve as best practice for designing an effective TRE [9]. The resultant six-dimensional approach enables an assessment of the context and results of data sharing efforts [9,11], including:

- **Safe people** Individuals who are trained, verified, authorised, and comply with ethical data usage and legal agreements are considered safe people. Only safe people are permitted access to requested data and code execution within a TRE, and their account information is not to be shared with another individual. For example, researchers who are approved by data custodians to work on projects that benefit the public would fall into this category.
- **Safe projects** Proposed projects must demonstrate appropriate use of data and justify the potential public benefit. Proposals will be reviewed by data custodians, and only approved projects can be conducted within a TRE.
- **Safe setting** Systems should store data securely and prevent unauthorised imports and exports of individual records, track researcher activity within the TRE for compliance monitoring, and provide tooling for running data analysis within the TRE. Two alternatives were presented in the original UKHDRA proposal to grant researchers remote access to a safe setting, (a) via a Virtual Desktop Interface (VDI) that requires users to authenticate before accessing view-only data and performing analysis, and (b) via an interface where only code execution can be run and tested against artificial (e.g., synthetic or transformed) datasets while hiding the actual data from the user. Ultimately, any necessary packages and tools needed to carry out a safe project should be deployable upon request.
- **Safe computing as an extension of safe setting** Safe settings imply on-premise hardware resources that data custodians are expected to provide. Given the demand for scalability, intense computation and robust security, cloud computing infrastructures are preferable today. Safe computing should provide a safe setting such that any outsourced private or public sector computing infrastructure should safeguard individual health records from unauthorised third-party access, including cloud provider administrators. Data in a safe setting or safe computing environment should also be encrypted and only be decryptable by the appropriate safe people.
- **Safe data** All identifying information should be removed from data within a TRE in a way that minimises risks of re-identification. Data should be standardised, and meta information about existing datasets within TREs, such as standards implemented, data descriptions, data origin, etc., should be made publicly available for greater discoverability and usability.
- **Safe outputs** Any data analysis outputs produced via a TRE should not be exported without proper authorisation, and, if authorised, only necessary results that support reporting or publishing should be exported. However, researchers may apply for the release of data or code from the TRE.
- **Safe return-an optional extension** Study subjects may consent to individual analysis results being returned to them. In this scenario, outputs produced by research users of TREs may return the corresponding data to the organisation or team that collected the subject data. The identifiers, if known, should only be available and re-identifiable at the original setting [11].

### 2.3. Workflow of Federated TREs

UKHDRA proposed their TRE best practice guidelines [9] based on existing efforts and experience building the ’Health Data Research Innovation Gateway’ (HDR Gateway) [12], which provides a central, searchable data directory. Through the HDR Gateway, data custodians submit metadata of their datasets, and researchers interested in the data can directly contact the data owners to request data access. The HDR Gateway also works with existing identity authentication systems used by institutions and trusted services. Leveraging the existing platform, UKHDRA plans to implement standardised application programmable interfaces (APIs) that can provide additional data access support with multiple TREs as extensions from the HDR Gateway.

**Figure 1:**
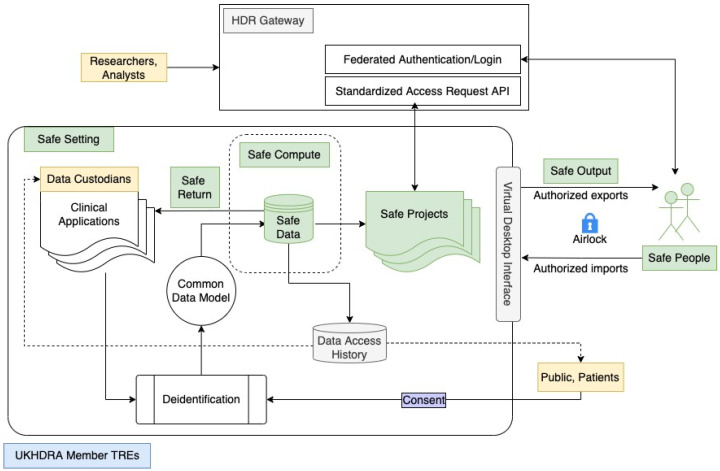
Overall diagram of a TRE and its integration with the HDR Gateway.

Figure 1 is adapted from the original concepts and more complex diagrams in the UKHDRA proposal [9]. It depicts in a simplified way the overall architecture of a typical TRE, the recommended interactions between its different components and the HDR Gateway, how the TRE abides by the ‘Five Safes’ framework with an extra ‘Safe Return’ extension, and how key stakeholders are involved in the workflow. UKHDRA member institutions (e.g., UK academic and NHS organisations) can implement an adaptor to the Gateway’s federated authentication/login portal (e.g., via OpenID Connect [13] and OAuth 2.0 [14]) that implements ‘Safe People’ requirements. Researchers and analysts can then be authenticated and easily verified (or pre-vetted) as ‘Safe People’. Using this existing federated authentication portal reduces the burden of user management within current and future TREs and prevents duplicated efforts. Additional implementations are required to achieve federated identity management across member institutions. For example, users should be uniquely identified across different organisations—the idea being similar to having unique and verifiable passports issued to users in a federation. Coupled with the federated credentialing process, TREs can also adopt Standardised Access Request APIs from the HDR Gateway to approve ‘Safe Projects’ with linkages to existing ‘Safe People’ accounts and handle data access requests directly.

Data custodians make data from their ‘Clinical Applications’ available to promote clinical research. In order to ensure that data accessed by ‘Safe Projects’ meet ‘Safe Data’ requirements, patients and anyone whose data are involved must provide consent to the safe use of their data, which is then deidentified to remove any personally identifiable information. To maximise the utility of the deidentified data, it is important that common data models or widely used ontologies are applied, such as the OMOP–Observational Medical Outcomes Partnership standards [15], to curate the ‘Safe Data’ that can then be accessed and meaningfully used by ‘Safe Projects’ within a range of federated TREs. ‘Safe Data’ either exist within the perimeter of data custodians (which is a ‘Safe Setting’ for the data) or is managed by trusted data providers with high-performance computing services that meet the ‘Safe Compute’ criteria. The history of data access should be logged within each member TRE as a key component of the ‘Safe Setting’. The history should be shared with the public and patients in addition to data custodians for reviewing and oversight purposes to maximise data use transparency.

It is worth noting that data accesses and analyses are executed within the ‘Safe Setting’ of each member TRE via ‘Beacons’, which are software tools that safely accept external data queries on ‘Safe Data’ and provide ‘Safe Return’ of results back to the original ‘Clinical Applications’ as needed. Should ‘Safe People’ require exporting of summary, other necessary data, or analytic code for publishing purposes or imports of additional data or programming tools into the TRE, they may do so via a secure interface such as the ‘Virtual Desktop Interface’ shown in Figure 1. To achieve federation, algorithms and tools used to support import and export into and out of the ‘Safe Setting’ should be standardised and reusable by other TREs. Any data export and import request that involves an external setting is subject to review and approval processes carried out by ‘airlock managers’. This is a security mechanism to ensure that only authorised imports and exports are allowed and thus satisfies the requirements of ‘Safe Output’.

### 2.4. TREs with a Stronger Community Involvement

Boniface et al. [16] proposed a Social Data Foundation (SDF) model for health and social care as a next-generation TRE with stronger community involvement. The SDF-variant expands from TREs that primarily support data sharing between conventional clinical databases (e.g., EHRs) to incorporate ‘social determinants’ of health. Social determinants of health are acquired from more diverse and modern data sources, such as lifestyle or behavioural data from wearable devices, environmental data from smart sensors, digital health platforms, and social media. There are two major components of SDF, (i) ‘stakeholder-sensitive data governance mechanisms’ that involve a wider range of stakeholders from the social and healthcare community throughout the decision-making lifecycle of the platform, and (ii) ‘datatrust services’ that provide functions to acquire diverse sources of data in the digital age and adhere to a progressive governance model that accommodates changes in research methods and stakeholder expectations over time. An example implementation of the SDF governance model leveraged decentralised technologies such as distributed ledgers and smart contracts [17] to manage the ownership and rights to data. This implementation provides stakeholders with a trustworthy environment for the secure sharing of a diverse range of data sources, along with a governance model that centres around the values and priorities of the broader social and healthcare community [16].

## 3. Benefits of TREs

The intended use of TREs is for securely sharing data and information across systems that have traditionally operated in disparate ways, all while protecting data privacy. Given the value of data in our information age, which is powered by data-intensive artificial intelligence and machine learning analyses and applications, the availability and wide adoption of TREs presents multi-faceted benefits to the public and healthcare participants, including, but not limited to, patients, providers, clinical and public health researchers, and healthcare planners and administrators. TREs can significantly improve the accessibility, quality and privacy of data, expedite data sharing, and help promote healthcare collaboration and advance medical and public health research. This section describes the benefits of TREs, which are also shared by PHTs (as we later explain in this article).

### 3.1. Data Accessibility

Despite limited access to data due to heterogeneous data storage and safekeeping practices, healthcare researchers, academic institutions, and industry alike have identified ways to support the data requirements of their research agendas. Through funding provided by government, enterprise, or other agencies, researchers have for decades conducted studies, such as clinical trials [18], where volunteers are recruited for the purpose of collecting data that are specific to meeting particular study objectives. Depending on the system that houses patient data and institutional directives, patients may be requested to provide their consent to their data being collected, deidentified, and released for research purposes during their first or each care visit. Data from patients whose consent is received may then be shared with researchers for secondary analysis [19]. Both approaches allow researchers to potentially collect data on the scale that is needed for supporting their preliminary findings and disseminating the results of their studies. The results are expected to be applicable to the population from which the analysed data originate, but their generalisability to a larger population is often difficult to test.

TREs can overcome this data access limitation by providing researchers with easier and relatively lower-cost access to a wider range of rich datasets needed and approved (ethically and otherwise) for their research studies. The enhanced accessibility of data should also motivate interested researchers and research entities who are underfunded to explore healthcare topics that are relevant to their respective fields, which in the long run could foster a more actively participating research community and drive medical discoveries and healthcare innovations forward more quickly.

### 3.2. Data Quality

The validity of statistical or analytical findings is conditioned upon using high-quality data sources. With the rapidly increasing use of automated and electronic data collection instruments nowadays, thanks to enabling technologies such as the Internet of Things and its connected sensors, healthcare data are becoming more abundant than ever. Growing numbers of health research institutions and government agencies around the world, especially in more developed countries, are also putting forth efforts to openly distribute anonymised or synthetic data to crowdsource novel insights on important problems. At the same time, the diversity, complexity, and enormous volumes of datasets available today come with more difficulty in validating the quality of those data within a limited timeframe [20]. It is often impractical for research teams, especially those who are more resource-constrained, to dedicate a tremendous amount of time and effort to assess the quality, integrity and usability of open data sources. Furthermore, publicly available datasets are often obsolete by the time they are released or can quickly become outdated, rendering them unusable by researchers, as many of these datasets were collected to primarily serve the purposes of public health surveillance or monitoring rather than the needs of medical and clinical research [21,22]. Even though the availability of large open datasets seems attractive (and is indeed so for some types of health research), the use of these data is generally less common in medical research due to the aforementioned data quality concerns.

The approach taken by TREs differs from open datasets in that they are promoted by official agencies and collaborating partners (in the United Kingdom), such as the NHS or the UKHDRA. Only accredited data sources are curated to form a data-sharing ecosystem with a trustworthy data and information governance model and standardised protocols and tooling for users. The combined components of a TRE are designed to help remove the overhead of manual data verification that would otherwise be performed by data requestors [9,23].

### 3.3. Data Privacy

As mentioned before, medical professionals and researchers have mutual concerns regarding the privacy and confidentiality of both patient information and their own intellectual property encoded as part of patient data. These concerns become major barriers to data sharing across institutions and research teams. TREs alleviate these concerns with built-in privacy-by-design provisions [24] of technologies and tools that focus on assuring the privacy and security of data access.

Firstly, control of data remains with the data custodian, and only those who satisfy specified requirements are able to interact with the TREs. Secondly, only data that are previously agreed upon as a necessary component of an approved project by approved personnel are made available through the TRE. Thirdly, the available data are not only deidentified but can also be minimised to remove any details that should be protected or kept private to the data owner. Data analysis and research outputs are carried out and processed within a secure environment without the risk of compromising the identity and privacy of patients. Fourthly, TREs include components that track and monitor the access and use of data (such as analyses performed on it), which ensures that sensitive and identifying information never leaves the controlled, secure environment. The recorded activity trail enables transparency and accountability within the environments and facilitates enhanced data surveillance for auditing, as well as reuse or replication of conducted analyses. Lastly, security checks are applied to additional tools, platforms, or other datasets newly introduced to TREs before they are incorporated into the environments. Overall, the assurance achieved by the rigorous requirements of TREs further builds the trust of patients that their data are used in a way that delivers public benefits without sacrificing their data privacy [6,23].

### 3.4. Expedited Data Sharing

Beyond data privacy concerns, a number of barriers exist in different dimensions that can delay or impede data sharing in healthcare. For example, data producers not being given appropriate credit for their contribution to data sharing practices creates ethical barriers and disincentives for data sharing; whereas the lack of compatible data formats, data standards, or sustainable technical solutions for data sharing has always been a stubborn technical challenge across healthcare information systems [22]. Although ethical issues are not entirely preventable, TREs can at least mitigate these concerns as data users are bound by regulations enforced within the TREs, and the data access and usage logs built into TREs can be traced back to the original source of data. Technical barriers, on the other hand, are more difficult to overcome because technical solutions, such as adding standardised data layers, require the direct involvement of a large group of participants. Compared to other efforts, TREs have more advantages in that they are sponsored and promoted by national (United Kingdom) institutions and healthcare policymakers, who are more capable of bringing together essential stakeholders and providing necessary guidelines for a wide range of TRE adopters nationwide to expedite the process of developing a standardised data sharing platform.

Another key aspect of TREs that speeds up the data sharing process is that they provide a ‘one-stop data shop’ or a ‘reference library’ for researchers, covering data and tooling for analytics, all within a single environment [6]. Having a single access point, data no longer need to be transferred to multiple sites, which not only expedites data access but also reduces duplication of data and storage overhead, as well as the risks of privacy breaches. As standards are being formalised and introduced into TREs, they are helping generate more liquidity, interoperability, and efficient deliveries of data [23].

### 3.5. Promoting Collaboration and Advancement in Healthcare Research and Practice

The design of TREs tackles the core requirements of health data sharing, and its successful implementation allows high-quality, trustworthy, diverse, extensive, and well-curated data to be shared with healthcare researchers in a timely, secure, privacy-preserving, and more equitable manner. The wide dissemination of data carries the potential of bolstering collaboration and leveraging the collective intelligence and experience of subject matter experts. In particular, TREs allow investigators focusing on similar research topics to form a more statistically significant or comprehensive dataset by joining data collected from diverse demographic or geographic populations, from which trends and patterns that may otherwise be overlooked can be observed. The collective insights thus obtained can aid in the development of more effective public health interventions. TREs also enable analytical results from safe projects to be relayed back to clinical experts, who can make more generalised interpretations about specific medical conditions. In cases where patients consent to being reidentified securely after the safe return of results, clinical researchers can leverage the combined data to promote personalised care.

In general, TREs can be seen as enablers helping us obtain a better understanding of the varying aspects of life sciences and medicine through more reproducible studies and more reliable research observations that can be utilised to enhance the safety and efficacy of medical diagnostics and disease treatments, drive healthcare innovations, and improve the overall quality of care for the public [23].

## 4. Select Examples of Successful TRE Implementations in the United Kingdom

A list of United Kingdom health and social care TREs is available via the HDR Gateway at [25]. Prominent on this list is the NHS Digital’s TRE service for England, which provides approved researchers with access to essential linked, deidentified health data covering millions of NHS health records and GP (General Practice) data assets. The TRE plays a pivotal role in answering important COVID-19-related research questions [26]. For example, one of its data resources involving a cohort of more than 54 million people in England (more than 96% of the English population) is being used to study the impact and effects of COVID-19 on cardiovascular diseases with regard to diagnosis, management and clinical outcomes, in order to benefit clinical care and public health, and inform healthcare policy [27]. Among the noteworthy published studies made possible by this TRE is one that looked into the association of COVID-19 vaccines with major venous, arterial, or thrombocytopenic events in a cohort of 46 million adults in England [28].

Another key health TRE, the United Kingdom Longitudinal Linkage Collaboration (UK LLC) TRE, is also playing an important role in COVID-19 research. The TRE provides access to pre-pandemic and pandemic data from more than 20 longitudinal population studies linked with health and environmental data [29,30]. Also worth noting is Genomics England’s 100,000 Genomes Project TRE, which offers access to deidentified genomic sequences, variants, and genes from over 100,000 genomes, in addition to clinical and omics data. The TRE features a virtual desktop environment with the most up-to-date research data, a wiki-based platform and chat facility to facilitate communication between researchers, command-line tools to perform analyses, and access to Genomics England’s high-performance AWS (Amazon Web Services) compute cluster [31]. The TRE is serving approved researchers working on a variety of topics [32], including COVID-19 vaccines and treatments [33].

In Wales, the SAIL (Secure Anonymised Information Linkage) Databank TRE supports research that is aimed at improving health, well-being, and public services. In addition to health data, SAIL’s data assets include a range of administrative datasets that can be used to build rich longitudinal research cohorts [34,35]. In Scotland, there are four TREs known as ‘regional safe havens’ dealing with local health data in Aberdeen, Dundee, Edinburgh, and Glasgow, in addition to a ‘national safe haven’ for all Scottish national health data [36]. The Honest Broker Service is the equivalent TRE for Health and Social Care (HSC) in Northern Ireland [37].

Finally, no survey of health-related TREs would be complete without a mention of the United Kingdom ONS Secure Research Service (SRS), which provides access to a wealth of health and other data assets [38]. An online ‘ONS SRS Metadata Catalogue’ allows users to conveniently search and retrieve metadata about these assets [39]. A useful list of other United Kingdom Digital-Economy-Act-(DEA)-2017-accredited TREs is available via the United Kingdom Statistics Authority at [40].

To prevent duplication of effort and of information governance and to further minimise privacy breach risks, the Goldacre Review [7] recommends that for any existing environment providing access to a given collection of datasets (such as the above-mentioned TRE examples), the environment should be properly catalogued, advertised, and reused rather than recreated and duplicated for any future analyses involving the same collection of datasets.

## 5. PHTs: Key Concepts, Benefits and Examples

**Figure 2:**
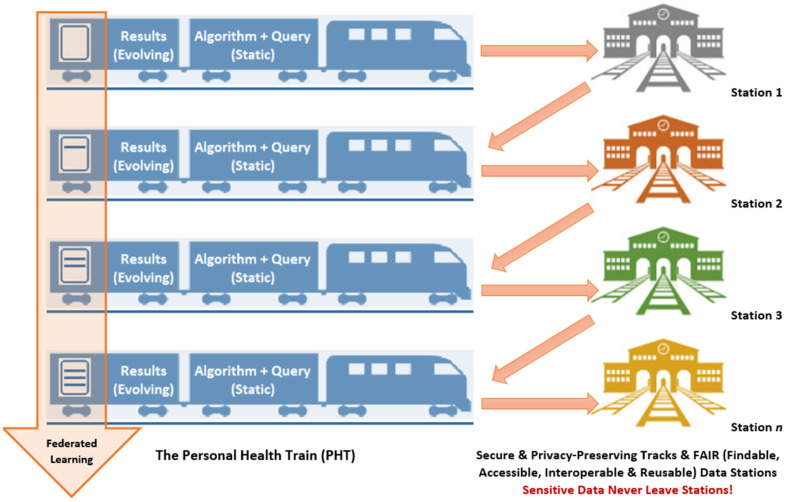
Key components of a PHT environment. Data owners control what a visiting ‘train’ is allowed to do with their data. Each ‘data station’ implements its own set of house rules that define what ‘trains’ can do whilst visiting. ‘Stations’ can range from very large databases to small personal lockers containing the data of one individual. Note how federated learning from data evolves as the ‘train’ (research question) moves from one ‘data station’ to the next.

Pioneered by a network of public and private health, healthcare and biomedical research stakeholders from mainland Europe (mainly in the Netherlands and Germany), PHTs were conceived to enable healthcare professionals and researchers to work with, and combine, health data from distributed sources or institutions (‘data stations’), providing them with responsible data access for distributed analytics while addressing the privacy protection concerns and needs of data owners, including individual patients and citizens. Privacy protection is imperative to earning and maintaining data owners and the public’s trust in medical and health research and ensuring their continuing engagement and contribution of vital data. PHT data stations adopt the FAIR principles, FAIR being an acronym referring to ‘Findable, Accessible, Interoperable and Reusable’ data that are amenable to concerted analyses. The PHT ‘train’ metaphor is used to illustrate the concept of federated (decentralised) data analysis. The essence of the PHT approach is that the research question (train) travels to the data (in FAIR data stations) rather than data from various sources having to be transported to the research question [8,41,42,43,44] (Figure 2).

Like TREs, PHTs adopt privacy-by-design principles [24] to protect personal health data, whereby sensitive data never leave their providing institutions or data stations (individual hospitals, clinical centres, cancer registries, etc., in the same or in multiple countries). The analysis is always brought to the data, allowing the data to remain at the source at all times [43,44]. PHTs are thus very similar (in their general concept) to the ‘software agents’ privacy-preserving approach proposed by Kamel Boulos et al. back in 2005/2006 [45], but with the explicit addition of the notion of ‘daisy-chained’ data stations. 

Model PHT architecture and implementation details (Tübingen implementation of the PHT) are available at [46,47]. Alternative PHT implementations include PADME (Platform for Analytics and Distributed Machine Learning for Enterprises), developed by a team at FIT (Fraunhofer Institute for Applied Information Techniques, Sankt Augustin, Germany) and other academic centres in Germany [48], and vantage6 (priVAcy preserviNg federaTed leArninG infrastructurE for Secure Insight eXchange), an open source PHT implementation for federated learning and discoveries from distributed FAIR data stations that was developed by The Netherlands Comprehensive Organisation–Integraal Kankercentrum Nederland (IKNL) [49].

Besides data privacy, PHTs share the same other benefits afforded by TREs, though with a more pronounced emphasis on federated (evolving) learning and discoveries from distributed multicentre data as the research train (questions) transits and the corresponding results (answers) progress, or build-up, from one data station to another [50,51] (Figure 2). PHTs offer a promising approach for conducting multicentre, large-scale medical and health research in an efficient and privacy-preserving manner, as demonstrated, for example, by Deist et al. [51] in their cancer survival prediction modelling study involving data from more than 20,000 lung cancer patients spanning eight oncology institutes in five countries, which they were able to complete in just four months. Deist and colleagues rightly describe their PHT-powered study as “machine learning without sharing patient data, quickly and at scale” [51].

Additional examples of healthy living, health and healthcare research use cases and projects harnessing PHT infrastructures are available at [52,53,54,55] and cover various topics, such as studying patterns of incidence and survival of rare cancers in Europe and Asia, predicting the best treatment for patients with hip or knee arthritis, supporting treatment decisions in mental health, finding answers to clinically relevant questions to improve the quality of care for cardiac patients, and many more.

## 6. Future Directions

Looking ahead, a hybrid environment combining both TRE and PHT approaches by bringing the federated learning train concept to TREs would be extremely useful [56]. Such an environment would enable federated learning from multiple TREs, e.g., across the different NHS TREs in England, Wales, Scotland, and Northern Ireland. Moreover, whilst the PHT decentralised data approach is already excellent, there is room for further developments during the coming decade with the introduction of Web3 and its privacy-preserving technologies, subject to these newer technologies gaining wide user acceptance and properly mitigating any associated security or other risks. Web3 builds on blockchain protocols to support the development of decentralised Web applications and enable users to control their own identity, content, and data, e.g., control access to their individual health wearable data held in small personal lockers, in a decentralised, peer-to-peer trust model [57].

## 7. Conclusions

Privacy-by-design (or data-protection-by-design) research environments, namely TREs and PHTs, can mitigate harmful data sharing and linking risks and help increase public trust in research involving personal and other sensitive data. They are becoming increasingly important for conducting large-scale privacy-preserving health research and for enabling federated learning and discoveries from big healthcare datasets spanning multiple institutions, as evidenced by the examples provided in this article of successful implementations of these environments (especially in the United Kingdom) and of large-scale studies that used them. The privacy-by-design principles and general architectures of these environments should also prove useful in other countries beyond the United Kingdom and continental Europe.

## Data Availability

Not applicable.
